# The complete chloroplast genome sequence of medicinal plant *Centipeda minima* (Compositae)

**DOI:** 10.1080/23802359.2022.2032437

**Published:** 2022-02-03

**Authors:** Xianheng Ouyang, Anliang Chen, Yangjun Mao, Kui Zhang, Peng Gui, Yang Yang, Luying Zuo

**Affiliations:** a School of Forestry and Biotechnology, Zhejiang A&F University, Hangzhou, China; b School of Pharmaceutical Sciences, Hunan University of Medicine, Huaihua, China

**Keywords:** Chloroplast genome, *Centipeda minima* (L.) A. Braun et Aschers (Compositae), Phylogenetic

## Abstract

The complete chloroplast genome sequence of *Centipeda minima* (L.) A. Braun et Aschers (Compositae) has been characterized by Illumina pair-end sequencing. The chloroplast genome is 152,351 bp in length, including a pair of inverted repeats (IRs) of 25,004 bp (12.1%) and a large single copy (LSC) and a small single copy (SSC) region of 83,972 bp (55.1%) and 18,371 bp (32.8%), respectively. The chloroplast genome includes 127 genes, which contain 83 protein-coding genes, 36 transfer RNA genes, and 8 ribosomal RNA genes. The nucleotide composition is asymmetric (31.1% A, 18.5% C, 19.0% G, 31.4% T). The overall GC content of *C. minima* chloroplast genome is 37.5%. Phylogenetic analysis illustrates that *C. minima* is closely related to other Asteraceae species, including *Helianthus annuus* subsp*. texanus*, *Tithonia diversifolia* and *Xanthium sibiricum* with a strong bootstrap value of 100.

*Centipeda minima* (L.) A. Braun et Aschers (Compositae) has long been used to treat nasal allergy, diarrhea, asthma, and malaria in the practice of traditional Chinese herbal medicine (Li et al. 2020; Jia et al. 2021). However, there is no study about the complete chloroplast genome of *C. minima*. In our study, we characterized the complete chloroplast genome sequence of *C. minima* by Illumina pair-end sequencing. The chloroplast genome information in our study will have significance for further research on phylogenetic relationships and conservation of *C. minima*. The annotated genome sequence was submitted to GenBank (accession number: MZ169540.1).

The sample of *C. minima* was collected from Zhejiang province, China (Zhejiang A&F University:119° 73′E, 30° 26′N) and a specimen was deposited at the Herbarium of Zhejiang A&F University (ZJFC) under the voucher number of OY01, Shuihu Jin (jsh501@163.com). There was no endangered or protected species involved in the study, and no specific permissions were required for the sample. Additionally, this study was supported by the key Research and Development Program of Zhejiang Province [No. 2019C02024]. We confirm that the locations are not privately owned or otherwise protected. The processes of extracting the total DNA and Illumina Hiseq 2500 sequencing was accomplished by Biomarker Technologies, Inc. (Beijing, China). We trimmed and assembled raw data using the MITObim v1.8 program (Hahn et al. 2013) and annotated the cp DNA sequence with program GENEIOUS R8.0.2 (Biomatters Ltd., Auckland, New Zealand) using the chloroplast genome of *Xanthium sibiricum* (NC_042232.1) as reference (Somaratne et al. 2019).

The complete chloroplast genome is 152,351 bp in length, including a pair of IRs of 25,004 bp (12.1%) and an LSC and an SSC region of 83,972 bp (55.1%) and 18,371 bp (32.8%), respectively. It consists of 127 genes, containing 83 protein-coding genes, 36 transfer RNA genes, and 8 ribosomal RNA genes. Within the *C. minima* chloroplast genome, 8 genes (*atpF*, *ndhA*, *ndhB*, *petB*, *rpl2*, *rpoC1*, *rps16*, and *ycf2*) contained a single intron, and 3 genes (*clpP*, *rps12*, and *ycf3*) contained 2 introns. The nucleotide composition is asymmetric (31.1% A, 18.5% C, 19.0% G, 31.4% T), and the overall GC content in the *C. minima* chloroplast genome is 37.5%.

Based on complete chloroplast genome sequences from the National Center for Biotechnology Information, the phylogenetic of *C. minima* combines with 12 Asteroideae species and 1 Campanulaceae species as outgroups. Sequences were aligned using MAFFT v7.017 (Katoh and Standley 2013). The best-fitting substitution model (GTR + G) was inferred using Modeltest 3.7 (Posada and Buckley 2004), and phylogenomic relationships were reconstructed with maximum likelihood (ML) using MEGA X (Kumar et al. 2018). To evaluate the supporting values of each node, 1,000 bootstrap replicates were conducted (Figure 1). The tree illustrates that *C. minima* is closely related to other Asteraceae species, including *Helianthus annuus* subsp*. texanus, Tithonia diversifolia* and *Xanthium sibiricum* with a strong bootstrap value of 100. The *C. minima* complete chloroplast genome in our study will provide useful data for subsequent research on phylogeny, DNA barcoding, and conservation genetics.

**Figure 1.**
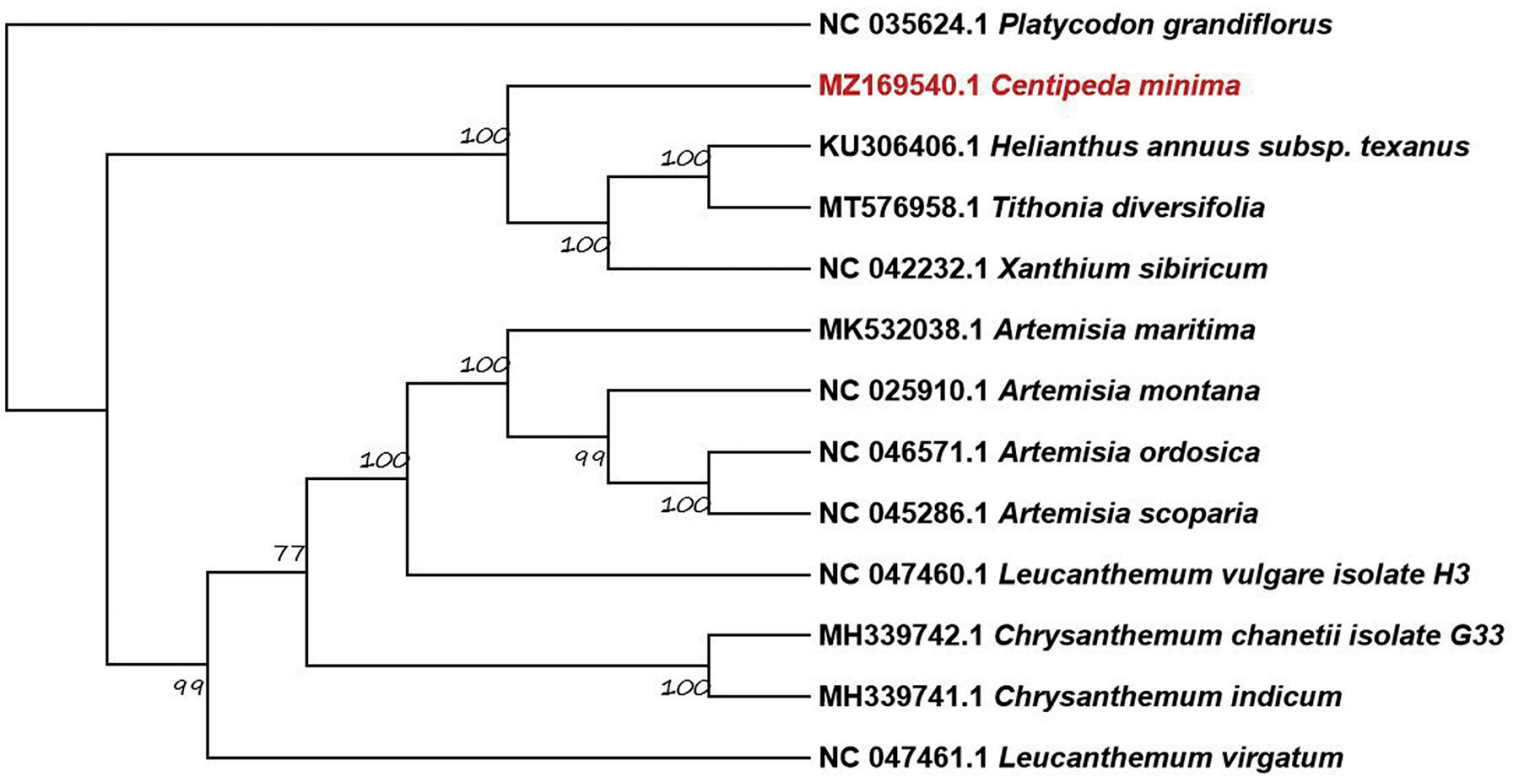
Phylogram inferred from the complete chloroplast genome sequences.

## Author contributions

**Xianheng Ouyang**: Methodology, Data curation, Formal analysis, Software, Writing- manuscript. **Anliang Chen**: Methodology, Funding acquisition. **Yangjun Mao**: Methodology**, Kui Zhang**: Methodology, **Peng Gui**: Investigation, **Yang Yang:** Investigation, **Luying Zuo**: Methodology. All authors agreed to the published version of the manuscript.

## Data Availability

The genome sequences of this study is openly available in GenBank (accession number MZ169540.1, https://www.ncbi.nlm.nih.gov/nuccore/MZ169540.1/). The associated BioProject, BioSample, and SRA number are PRJNA753010 and SAMN20669373, and SRR15384391 respectively.
